# Supplementary material for: Single dose of chimeric dengue-2/Zika vaccine candidate protects mice and non-human primates against Zika virus
Source: Nat Commun. 2021 Dec 16;12:7320. doi: 10.1038/s41467-021-27578-w (PMC8677809; doi:10.1038/s41467-021-27578-w)
Supplement: Supplementary file 1 — Supplementary Information [file 41467_2021_27578_MOESM1_ESM.pdf]

Supplementary Information

Single dose of chimeric dengue-2/Zika vaccine candidate protects mice and non-human primates against Zika virus

Whitney R. Baldwin[1,2], Holli A. Giebler[1,2], Janae L. Stovall[1], Ginger Young[2], Kelly J Bohning[2], Hansi J. Dean[2], Jill A. Livengood[1,2], Claire Y.-H. Huang[1*]

[1]Arboviral Diseases Branch, Division of Vector-Borne Diseases, Centers for Disease Control and Prevention, Fort Collins, Colorado, USA

[2]Takeda Vaccines Inc., Cambridge, MA, USA

Supplementary Table 1. SNV frequencies (≥10%) in P5 and P10 samples[a] of D2/ZK-V4

Gene	Nucleotide Position	Change	AA Change[b]	P5A	P10A	P5B	P10B
prM	474	U→A	Ser→Arg		69.10		
	535	U→G	Leu→Val			25.76	73.86
	536	U→C	Leu→Ser	50.6			
	614	U→C	Met→Thr	22.7			
	614	U→G	Met→Arg			24.73	15.58
	746	C→U	Ser→Phe			15.01	
E	1537	G→A	Gly→Ser				62.84
	1538	G→A	Gly→Asp		29.60		
	2155	C→U	His→Tyr				16.23
NS1	3261	A→U					13.00
NS3	5001	A→G				11.35	
	6364	A→G	Ile→Val			26.30	15.05
NS4A	6710	A→G	Tyr→Cys				65.96
NS4B	6912	A→G			10.74		17.08
	6976	U→G	Ser→Ala		76.62		14.95
NS5	7619	C→U	Thr→Ile				10.18
	7842	A→G				16.82	67.07
	8073	U→C					11.25
	8289	C→A			10.53		
	8589	C→U		30.9	14.65		
	10224	U→C					11.71
3'UTR	10503	G→U					11.03
	10649	C→U			35.28		

[a] Results from independent lineages (A and B) are shown.
[b] Blank indicates synonymous change or change in UTR.

Supplementary Table 2. SNV frequencies (≥10%) in P5 and P10 samples[a] of D2/ZK-V5

Gene	Nucleotide Position	Change	AA Change[b]	P5A	P10A	P5B	P10B
prM	474	U->A	Ser-Arg			58.45	
	536	U->C	Leu-Ser			16.07	92.22
	670	A->G	Thr-Ala	59.49	98.82		
	770	C->U	Thr-Met				57.16
	775	U->C	Ser-Pro		68.94		
E	1204	A->C	Lys-Gln	10.78			
	2274	A->C				57.66	12.57
	2341	U->A	Phe-Ile	60.42	98.93		
NS1	3225	A->C	Gln-His			19.57	87.71
NS2A	4088	C->U	Thr-Ile				14.78
NS3	5517	U->C				55.81	10.95
NS4B	6912	A->G			20.27		21.61
	6967	C->A	Arg-Ser	11.36			
	7181	C->U	Pro-Leu			18.22	84.63
3'UTR	10499	GG->CU			11.79		12.23
	10649	C->U		12.20	40.37		38.96

[a] Results from independent lineages (A and B) are shown.

[b] Blank indicates synonymous change or change in UTR.

Supplementary Table 3. SNV frequencies (≥ 10%) in P5 and P10 samples[a] of D2/ZK-V5-Pr

Gene	Nucleotide Position	Change	AA change[b]	P5A	P10A	P5B	P10B
5'UTR	**57***	C->T	5'UTR*		34.05		
PrM	770	C->T	Thr-Met	98.18	99.56		
	775	T->C	Ser-Pro				11.70
	913	A->G	Ile-Val			15.68	31.89
E	1147	A->G	Ile-Val			16.70	23.17
	1404	G->A				17.86	13.32
	1470	T->C				18.28	22.43
	1537	G->A	Gly-Ser				14.57
NS1	**2624***	A->G	Asp-Gly*		3.36		
	2913	T->C				15.50	30.61
NS3	4858	T->C					11.09
	5199	T->C					17.00
	**5315***	T->A	Val-Glu*				12.19
NS4B	6912	A->G			15.94		
	6976	T->G	Ser-Ala			24.14	22.90
	7225	G->T	Ala-Ser			15.09	32.69
	7539	C->T		97.95	99.67		
	7583	C->T	Ser-Phe	98.14	99.07	28.32	40.20
NS5	8134	A->T	Asn-Tyr				14.92
	9244	C->T		15.12			
3'UTR	10499	GG->CT			10.01		
	10649	C->T			35.51		29.59

[a] Results from independent lineages (A and B) are shown.
[b] Blank indicates synonymous change or change in UTR.
*Attenuation loci of DENV-2 PDK-53: any SNV detected at ≥1% frequency are indicated for these loci

Supplementary Table 4. Primers used for confirmation Sanger sequencing of chimeric D2/ZIKV

cDNA Fragment	Primers for RT-PCR		Primers for sequencing	
	Name	Sequence	Name	Sequence
Frag 1	D2-1	AGTTGTTAGTCTACGTGGACCGAC	D2-24	AGTTGTTAGTCTACGTGGACCGAC
	cZK-1416	GGGAGCCATGAACTGACA	ZK-536	GGCCATATCTTTTCCAACCACATT
			ZK-1025	AGGTGGGACTTGGGTTGATGTTG
			cZK-538	GCCTCCCCAGCATCGTTTCT
			cZK-941	GTATATGACTTTTTGGCTCGTT
			cZK-1416	GGGAGCCATGAACTGACA
Frag 2	ZK-1379	TCTGGAGTACCGGATAATGCTGTC	ZK-1379	TCTGGAGTACCGGATAATGCTGTC
	cD2-2688	CATGATTCCTTTGATGTCTCCTGTC	ZK-1900	GTACTGCAGCGTTCACATTCACCA
			cZK-1785	CAAGGGCCGTGTGAACTGCTC
			cZK-2240	CATTCTCTTGGCACCTCTCACAG
			cD2-2670	CTCCTGTCATAATAGTTAACTTCACC
Frag 3	D2-2585	GTGGAATCCGCTCAGTAACAACAC	D2-2625	GTAACAAGACTGGAGAATCTGATGTGG
	cD2-4323	GGGCTGCTTCCTGATATYTCTGCC	D2-3111	GCCACTGGCCAAAATCACACACC
			D2-3589	GAAACATGCAATACTACTAGTTGCAG
			cD2-3050	CAATGAAAGAGGCTTTCTCTATCTTCC
			cD2-3523	CTAAGCATTTCCTCCAGGAACAATGC
			cD2-4043	CGTCAATGCTAATGGTATCCAATCTG
Frag 4	D2-3986	CAAAACGCATGGAAAGTGAGTTGCAC	D2-4005	GTTGCACAATATTGGCAGTGGTGTCC
	cD2-5342	GATAATCAGGTTGTAGTTTGGCACTC	D2-4520	CCTGTGGGAAGTGAAGAAACAACG
			D2-4996	GGTAATGGTGTTGTTACAAGGAGTG
			cD2-4462	CTGCCGTGATTGGTATTGATACAGG
			cD2-4913	TGGAGATCCTGACGTTCCAGGAG
			cD2-5342	GATAATCAGGTTGTAGTTTGGCACTC
Frag 5	D2-5305	GGGAGATTGTGGACCTAATGTGTC	D2-5312	TGTGGACCTAATGTGTCATGCCAC
	cD2-6736	GGTGTTCTCTGTTTTTCAGGTTCTGG	D2-5831	CATGAAACCAGTCATACTAACAGATGG
			D2-6314	AATTGAAACCCAGATGGTTGGATGC
			cD2-5772	GGTCTATAACCCTCTCAGCCTTG
			cD2-6249	CTTTTGTCCAGATTTCAACTTCCACG
			cD2-6729	CTCTGTTTTTCAGGTTCTGGAATAAGC
Frag 6	D2-6667	ACGGCTAGCATCCTCCTATGGTACG	D2-6687	GTACGCACAAATACAGCCACACTGG
	cD2-8041	GTTAAGGACTCTGAGTGTTCGTCCTGC	D2-7154	CAACCCCATAACTCTCACAGCAGC
			D2-7630	TGGAAAAGCCGATTGAACGCATTGG
			cD2-7101	AGTAGCATCCAATGGCGAGAAGG
			cD2-7570	CGTCTCTCCTATGTTGCCAGTTCC
			cD2-8030	TGTTCGTCCTGCTTCCACTGTGG
Frag 7	D2-7990	GTTTTCTTCATCCCGCCAGAAAAGTGTG	D2-8012	GTGTGACACATTATTGTGTGACATAGG
	cD2-9382	GGTGAAAGTATTGAGTCCATAGGTGCC	D2-8428	GAGCATGAAACATCATGGCACTATGACC
			D2-8957	TGGGAAAAAGAGAGAAGAAGCTAGG
			cD2-8428	CATAGTGCCATGATGTTTCATGCTC

			cD2-8890<br>cD2-9365	GTTTCACACTTTCCTTCAAGATGGAG<br>GGTGCCAACTTGTCCACTACCTC
Frag 8	D2-9310<br>cD2-10695	CGTACCAAAACAAGGTGGTGCGTGTGC<br>AGAACCTGTTGATTCAACAGCACCATTCC	D2-9323<br>D2-9782<br>D2-10249<br>cD2-9727<br>cD2-10185<br>cD2-10686	GTGCGTGTGCAAAGACCAACACC<br>CCAAGATGAACTGATTGGCAGAGC<br>CCATGAAAAGATTCAGAAGAGAAGAGG<br>TGGAACAACGAGTACGCGACCGTC<br>CTGTGTATTCTTCATTGCCTATAAGG<br>TCAACAGCACCATTCCATTTTCTGG

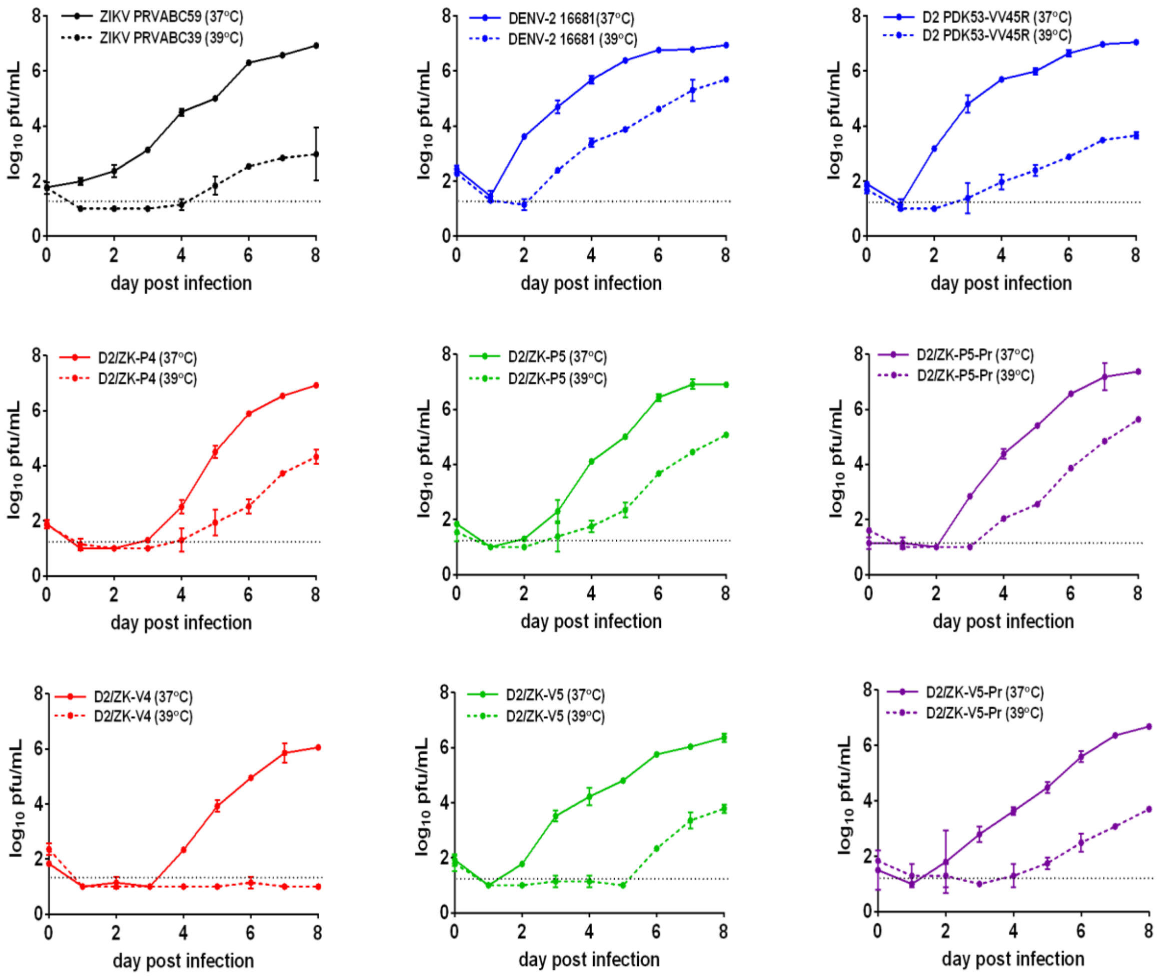

Supplementary Figure 1. Growth kinetics of viruses in LLC-MK$_2$ cells at 37°C and 39°C (Evaluation of temperature sensitivities of the viruses) represented as the mean of n=2 samples from independent culture flasks. Error bars represent the standard deviation. Dashed lines indicate the limit of detection (LOD) of the plaque titration assay. Negative samples were assigned half value of the LOD for GMT determination of each duplicate sample.

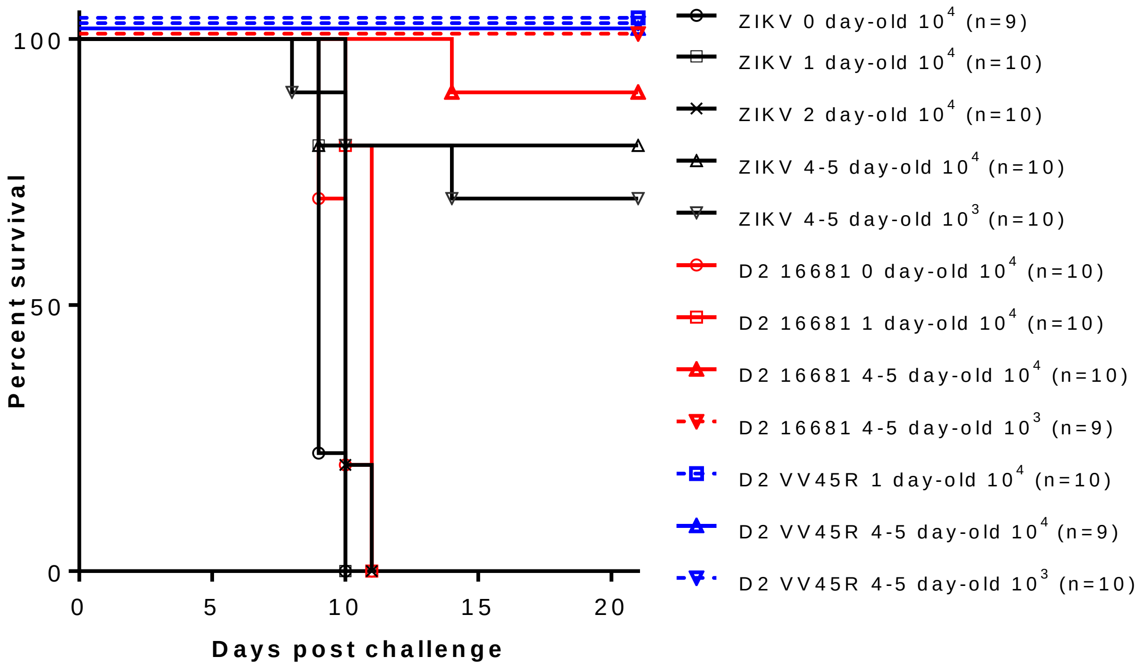

Supplementary Figure 2. Mouse neurovirulence model for ZIKV PRVABC59 and DENV-2 16681. Newborn CD-1 mice (0- to 5-day-old) were i.c. challenged with 10^3 or 10^4 pfu of wt ZIKV, wt DENV-2, or D2 PDK-53 VV45R vaccine. Most 4–5-day-old mice survived after wt ZIKV and DENV-2 challenge. All mice under 2 days-old became morbid after 10^4 pfu of wt ZIKV or DENV-2 challenge. All mice (1-5 days-old) challenged by the D2 PDK-53-VV45R vaccine survived without sign of illness. Number (n) of mice used in each group is indicated in the symbol legend of the graph.

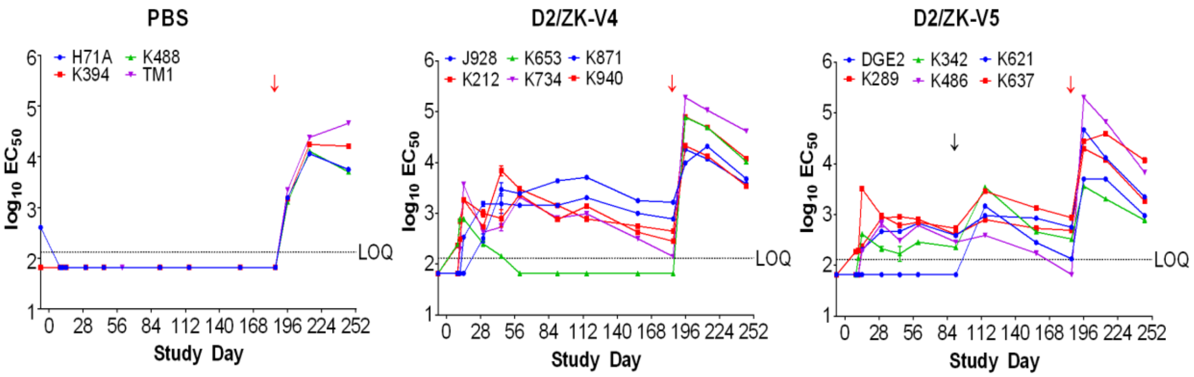

Supplementary Figure 3. Neutralizing antibody kinetics of rhesus macaques as measured by RVP assay. Black arrow indicates day of boost (D2/ZK-V5 group only) and red arrow indicates day of challenge. Day 30 and 45 samples were titrated in duplicate by 2 independent assays (n=2), and the mean with SD of the 2 assays were graphed. Samples from other study time points were titrated in duplicate from a single experiment (n=1). Negative samples were assigned half value of the assay LOQ (limit of quantification) for GMT determination.
