# Supplementary material for: Single dose of chimeric dengue-2/Zika vaccine candidate protects mice and non-human primates against Zika virus
Source: Nat Commun. 2021 Dec 16;12:7320. doi: 10.1038/s41467-021-27578-w (PMC8677809; doi:10.1038/s41467-021-27578-w)
Supplement: Supplementary file 3 — Reporting Summary [file 41467_2021_27578_MOESM3_ESM.pdf]

nature portfolio

Corresponding author(s): Claire Y-H.Huang

Last updated by author(s): Nov 19, 2021

Reporting Summary

Nature Portfolio wishes to improve the reproducibility of the work that we publish. This form provides structure for consistency and transparency in reporting. For further information on Nature Portfolio policies, see our Editorial Policies and the Editorial Policy Checklist.

Statistics

For all statistical analyses, confirm that the following items are present in the figure legend, table legend, main text, or Methods section.

n/a	Confirmed	
☐	☒	The exact sample size (*n*) for each experimental group/condition, given as a discrete number and unit of measurement
☐	☒	A statement on whether measurements were taken from distinct samples or whether the same sample was measured repeatedly
☐	☒	The statistical test(s) used AND whether they are one- or two-sided<br>*Only common tests should be described solely by name; describe more complex techniques in the Methods section.*
☐	☒	A description of all covariates tested
☐	☒	A description of any assumptions or corrections, such as tests of normality and adjustment for multiple comparisons
☐	☒	A full description of the statistical parameters including central tendency (e.g. means) or other basic estimates (e.g. regression coefficient) AND variation (e.g. standard deviation) or associated estimates of uncertainty (e.g. confidence intervals)
☐	☒	For null hypothesis testing, the test statistic (e.g. F, t, r) with confidence intervals, effect sizes, degrees of freedom and P value noted<br>*Give P values as exact values whenever suitable.*
☒	☐	For Bayesian analysis, information on the choice of priors and Markov chain Monte Carlo settings
☒	☐	For hierarchical and complex designs, identification of the appropriate level for tests and full reporting of outcomes
☒	☐	Estimates of effect sizes (e.g. Cohen's d, Pearson's r), indicating how they were calculated

Our web collection on statistics for biologists contains articles on many of the points above.

Software and code

Policy information about availability of computer code

Data collection	Standard software on equipment described in methods (ABI genetic analyzer, MiSeq sequencer, Celigo image cytometer, CFX-96 touch Real-time PCR system, and flourometer), and Microsoft 365 Excel were used for data collection and recording.
Data analysis	DNASTAR Lasergene 15: Sanger sequence analysis<br>Qiagen CLC Genomics workbench v.12: NGS analysis<br>GraphPad Prism 6.0 and 8.0: Data calculation, such as EC50 calculation, mean and standard deviation in figures, statistic analysis, and graphing<br>Microsoft 365 Excel: Data calculation (mean weight, average survival time, standard deviation) and experiment summary tables<br>R software (v 4.0.1, R Core team 2020): Estimation of limit of quantification for real-time RT-PCR assay. The code used with the software is developed by a US Geological Survey/Department of Interior and can be found at https://doi.org/10.5066/P9GT00GB

For manuscripts utilizing custom algorithms or software that are central to the research but not yet described in published literature, software must be made available to editors and reviewers. We strongly encourage code deposition in a community repository (e.g. GitHub). See the Nature Portfolio guidelines for submitting code & software for further information.

Data

Policy information about availability of data

All manuscripts must include a data availability statement. This statement should provide the following information, where applicable:

- Accession codes, unique identifiers, or web links for publicly available datasets
- A description of any restrictions on data availability
- For clinical datasets or third party data, please ensure that the statement adheres to our policy

All data generated and analyzed in this study are available in the main paper, supplementary information, and provided source data file.
Genomic sequence of the viruses used in this study are available in GenBank (https://www.ncbi.nlm.nih.gov/genbank) with following accession codes: U87411 for DENV-2 16681, U87412.1 for DENV-2 PDK-53, KU321639 for ZIKV Brazil SPH2015, and KX377337 for ZIKV PRVABC59

Field-specific reporting

Please select the one below that is the best fit for your research. If you are not sure, read the appropriate sections before making your selection.

☒ Life sciences ☐ Behavioural & social sciences ☐ Ecological, evolutionary & environmental sciences

For a reference copy of the document with all sections, see nature.com/documents/nr-reporting-summary-flat.pdf

Life sciences study design

All studies must disclose on these points even when the disclosure is negative.

Sample size

Sample sizes were based on similar studies previously conducted and reported (listed below). In addition, animal samples sizes were also reviewed and approved by statistician serving on the IACUC committee to review sample size and approve the animal protocol for the study.
1. Huang, C. Y., Silengo, S. J., Whiteman, M. C. & Kinney, R. M. Chimeric dengue 2 PDK-53/West Nile NY99 viruses retain the phenotypic attenuation markers of the candidate PDK-53 vaccine virus and protect mice against lethal challenge with West Nile virus. J Virol 79, 7300-7310, doi:10.1128/JVI.79.12.7300-7310.2005 (2005).
2. Huang, C. Y. et al. Dengue 2 PDK-53 virus as a chimeric carrier for tetravalent dengue vaccine development. J Virol 77, 11436-11447, doi:10.1128/jvi.77.21.11436-11447.2003 (2003).
3. Butrapet, S. et al. Attenuation markers of a candidate dengue type 2 vaccine virus, strain 16681 (PDK-53), are defined by mutations in the 5' noncoding region and nonstructural proteins 1 and 3. J Virol 74, 3011-3019, doi:10.1128/jvi.74.7.3011-3019.2000 (2000).
4. Huang, C. Y. et al. Genetic and phenotypic characterization of manufacturing seeds for a tetravalent dengue vaccine (DENVax). PLoS Negl Trop Dis 7, e2243, doi:10.1371/journal.pntd.0002243 (2013).
5. Dietrich, E. A., Ong, Y. T., Stovall, J. L., Dean, H. & Huang, C. Y. Limited Transmission Potential of Takeda's Tetravalent Dengue Vaccine Candidate by Aedes albopictus. Am J Trop Med Hyg 97, 1423-1427, doi:10.4269/ajtmh.17-0185 (2017).
6. Young, G. et al. Complete Protection in Macaques Conferred by Purified Inactivated Zika Vaccine: Defining a Correlate of Protection. Sci Rep 10, 3488, doi:10.1038/s41598-020-60415-6 (2020)
7. Osorio, JE, J.N. Brewoo, S.J. Silengo, J. Arguello, I.R. Moldovan, M. Tary-Lehmann4, T.D. Powell, J.A. Livengood, R.M. Kinney, C.Y.-H. Huang, and D.T. Stinchcomb (2011) Efficacy of a Tetravalent Chimeric Dengue Vaccine (DENVax) in Cynomolgus Macaques. Am. J. Trop. Med. Hyg 84, 978-987, doi:10.4269/ajtmh.2011.10-0592 (2011)

Data exclusions

No data were excluded from analyses

Replication

All attempts in replication were successful as described below:
1. Cell based in vitro experiments were conducted with at least 2 independent biological replicates for each sample (Fig 2a-b and Fig 3a-c). Two independent experiments were conducted to complete all viruses in Fig 2a. The 2 experiments showed reproducible outcomes of 4 virus groups (wt ZIKV, DENV-2, D2/ZK-P5, and -V5; one experiment with independent triplicate, and the other with duplicate for each virus). Other studies conducted in single experiment always include DENV-2 16681 and DENV-2 VV-45R as controls which have been previously studied frequently in same type of experiments. Reproducible results of these 2 control viruses were checked to ensure the current experiment correlated well with previous study.
2. Mosquito studies (Fig 4a-d) required multiple experiments to achieve desired sample size for the study. Replicate experiments of the DENV-2 16681 and DENV-2 VV45-R have been conducted and published. Both viruses were included in this study as controls, and similar results were obtained from experiments in this study indicating successful replication of experiments.
3. The CD-1 mouse study (Fig 5) was conducted in 2 separate experiments, each with a wt ZIKV as control to bridge the two experimental results. Reproducible results of the control were obtained from the 2 experiments.
4. Single and double dose efficacy evaluation in AG129 (Fig 6) was initially conducted in a single experiment for the D2/ZK-V5 candidate only. The result show clear protection effect of the V5 candidate, and single dose appeared to be as effective as double immunization. This single experiment supports full evaluation of all 3 candidates in replicate experiments shown in Fig 7.
5. The full efficacy evaluation with all 3 vaccine candidates (Fig 7) in AG129 mice included PBS (naive control), D2/ZK-V4, -V5, and -V5-Pr vaccine groups, and 3 parental chimera counterparts groups. Two independent experiments were required to complete the full study, and 3 groups (PBS, V4 and V5) were replicated in both experiments. Reproducible outcomes of these viruses were observed in these independent experiments.
6. All samples obtained from above experiments were tested in replicates (duplicate or triplicate depending on assay types). Independent assays were not conducted for all samples due to limit of sample volume,but positive and negative controls were included in all assays to validate assay accuracy and reproducibility. For Fig 8b R-mFRNT results, 2 independent assays were conducted for all NHP samples (larger volume permitting replicative experiments) and each sample was titrated in triplicate/experiment. Highly reproducible results of all samples

	were obtained from the independent experiments with low SD, and both results were included for final analyses.
Randomization	Allocation of animals (AG129 and NHP) into different groups were random, with exception that each group contained both male and female (in separated cages) to ensure similar sex proportion among all groups.<br>The CD-1 dams were randomly assigned to different groups, and the newborn litter of each dam was maintained in the same group (cage) with their mother. For the groups with pups under desirable numbers, pups from other litters (with extra numbers) were added into the group before experiment to even out the pup numbers among the groups whenever possible.<br>Mosquitoes were also randomly allocated to different tested groups. All experiments were conducted using mosquitoes hatched from same generation of eggs of each colony.<br>For all other in vitro experiments of this study: randomization is not relevant for these in vitro studies. However, all experimental conditions were carefully controlled to ensure same experiment conditions were used for all groups. Positive and negative controls are always included in all experiments to ensure consistency among experiments.<br>Samples collected from all study groups described above do not require further randomization for assays, as they should be assigned to the same group from where they were obtained and randomization is not relevant.
Blinding	Investigators were not blinded during data collection or analysis because experiment planning, conducting, and analysis were typically performed by same personnel.

Reporting for specific materials, systems and methods

We require information from authors about some types of materials, experimental systems and methods used in many studies. Here, indicate whether each material, system or method listed is relevant to your study. If you are not sure if a list item applies to your research, read the appropriate section before selecting a response.

Materials & experimental systems

n/a	Involved in the study
☐	☒ Antibodies
☐	☒ Eukaryotic cell lines
☒	☐ Palaeontology and archaeology
☐	☒ Animals and other organisms
☒	☐ Human research participants
☒	☐ Clinical data
☐	☒ Dual use research of concern

Methods

n/a	Involved in the study
☒	☐ ChIP-seq
☒	☐ Flow cytometry
☒	☐ MRI-based neuroimaging

Antibodies

Antibodies used	Mouse mAb 4G2: CDC Lott# B82528-02-1; Provided by Arbovirus Reference Collection (ARC)/CDC. Used at 1:200 dilution for IFA in this study.<br>FITC-conjugated affiniPure goat anti-mouse IgG (H+L): Jackson Immunoresearch Lab, Inc. Cat # 115-095-062 Lot: 88785. Used at 1:15,000 dilution for IFA
Validation	Primary antibody, 4G2: a mouse mAb (IgG2a) originally developed by WRAIR and deposited to ARC/CDC for CDC internal research. It was made against DENV-2 New Guinea C strain in mice and is cross-creative to flaviviruses. Based on the product information provided by ARC, the lot was produced from non-infectious hybridoma cells and passed the release qualification validated by a Quality Assurance technician. In addition, this lot was produced in 2003, and has been used by investigators at CDC in ELISA, IFA, and PRNT against flaviviruses for years without any issues reported to ARC. In our lab, a positive control is used to validate and titrate the mAb before using it for any study. The development and characterization details of the mAb can be found in:<br>Am J Trop Med Hyg.1982:548-55. doi: 10.4269/ajtmh.1982.31.548.<br><br>FITC-conjugated affiniPure goat anti-mouse IgG (H+L): The antibody was validated by the commercial company. The validation can be found in the Lot specification sheet downloaded from https://www.jacksonimmuno.com/catalog/products/115-095-062. The antibody specificity validation statements are copied from the sheet and pasted below:<br>"Based on immunoelectrophoresis and/or ELISA, the antibody reacts with whole molecule mouse IgG. It also reacts with the light chains of other mouse immunoglobulins. No antibody was detected against non-immunoglobulin serum proteins. The antibody has been tested by ELISA and/or solid-phase adsorbed to ensure minimal cross-reaction with human, bovine, and horse serum proteins, but it may cross-react with immunoglobulins from other species."

Eukaryotic cell lines

Policy information about cell lines

Cell line source(s)	Vero WHO RCB 10-87: initially from WHO Reference Cell Bank and amplified to generate a seed stock that was used in the study. The Vero cells (CCL-81), LLCM-K2 cells (CCL-7), and C6/36 cells are available at ATCC.

Authentication	The seed stock amplified from Vero WHO RCB 10-87 used for the study has been used to generate Master Vero Cell Bank that was certified for TDV vaccine manufacture. No additional authentication was conducted for cell lines obtained commercially
Mycoplasma contamination	All cell lines were tested negative for mycoplasma contamination by PCR routinely
Commonly misidentified lines (See ICLAC register)	No commonly misidentified line was used in the study

Animals and other organisms

Policy information about studies involving animals; ARRIVE guidelines recommended for reporting animal research

Laboratory animals	Time pregnant CD-1 mice and their pups (0- to 5-day old). AG129 mice: 3-5 weeks old, mixed sex. Indian rhesus macaques: 1-3 years old, mixed sex. Mosquitoes: 4-6 day-old Aedes aegypti (2012 Poza Rica colony) and Aedes albopictus (Lake Charles and ELG colonies)
Wild animals	No wild animals were used in this study
Field-collected samples	No field-collected samples were used in this study
Ethics oversight	The mouse study protocols were approved by the Institutional Animal Care and Use Committee (IACUC) at the Division of Vector-Borne Diseases, CDC (DVBD/CDC).<br>The study in non-human primates was reviewed and approved by the IACUCs at Alpha Genesis and DVBD/CDC.<br>An ethical approval is not required for use of mosquitoes.

Note that full information on the approval of the study protocol must also be provided in the manuscript.

Dual use research of concern

Policy information about dual use research of concern

Hazards

Could the accidental, deliberate or reckless misuse of agents or technologies generated in the work, or the application of information presented in the manuscript, pose a threat to:

No	Yes	
☒	☐	Public health
☒	☐	National security
☒	☐	Crops and/or livestock
☒	☐	Ecosystems
☒	☐	Any other significant area

Experiments of concern

Does the work involve any of these experiments of concern:

No	Yes	
☒	☐	Demonstrate how to render a vaccine ineffective
☒	☐	Confer resistance to therapeutically useful antibiotics or antiviral agents
☒	☐	Enhance the virulence of a pathogen or render a nonpathogen virulent
☒	☐	Increase transmissibility of a pathogen
☒	☐	Alter the host range of a pathogen
☒	☐	Enable evasion of diagnostic/detection modalities
☒	☐	Enable the weaponization of a biological agent or toxin
☒	☐	Any other potentially harmful combination of experiments and agents
